# Structural characterization, rheological properties, and skin bioactivities of a glucomannan from *Bletilla striata*

**DOI:** 10.1007/s13659-026-00638-8

**Published:** 2026-06-23

**Authors:** Xuan Li, Qinghui Gu, Qile Hu, Tuo Deng, Lan Luo, Jianping Cai, Xudong Dong, Chunhua Wu, Mingyi Wu

**Affiliations:** 1 College of Material and Chemical Engineering, Southwest Forestry University, Kunming, 650224 China; 2 State Key Laboratory of Phytochemistry and Natural Medicines, Kunming Institute of Botany, Chinese Academy of Sciences, Kunming, 650201 China; 3 School of Chemical Science and Technology, Yunnan University, Kunming, 650500 China; 4 Shanghai Jiaxin Biotechnology Co., Ltd, Shanghai, 201108 China; 5 The First People’s Hospital of Yunnan Province, Kunming University of Science and Technology Affiliated Hospital, Kunming, 650032 China

**Keywords:** *Bletilla striata*, Glucomannan, Structure elucidation, Bioactivity

## Abstract

*Bletilla striata* is highly regarded in traditional Chinese medicine and has also gained widespread attention as a valuable medicinal plant resource. However, the molecular composition and bioactive potential of its polysaccharides present a significant knowledge gap. Herein, a specific glucomannan fraction, designated as BSP60, was purified from *Bletilla striata* tubers and subsequently subjected to structural, rheological, and biological evaluations. Comprehensive characterization via methylation-GC–MS, monosaccharide composition analysis, and 1D and 2D NMR techniques demonstrated that BSP60 possesses a β-(1 → 4)-linked backbone featuring alternating mannosyl and glucosyl units, with the mannose moieties exhibiting partial *O*-acetylation at their C-2 or C-3 positions (with an acetylation rate of 23.54%). Rheological studies showed that BSP60 aqueous solutions exhibit shear-thinning behavior and form an elastic-dominated viscoelastic network, with higher viscosity and storage modulus than dextran of comparable molecular weight, indicating enhanced intermolecular interactions. Biological assays revealed that the BSP60 effectively decreased the level of NO, IL-6, and TNF-α in LPS-triggered macrophages (RAW264.7). Furthermore, the scavenge ability of BSP60 to superoxide anion (O_2_^•−^) and ABTS^⁺•^ radicals are significantly higher than that of hyaluronic acid. This study systematically characterizes the structural features of glucomannan derived from *Bletilla striata*, thereby establishing a robust scientific basis for its expanded utilization in the development of biomedical materials and functional cosmetic ingredients.

## Introduction

The wide range of biological activity and diverse structural characteristics of polysaccharides extracted from natural medicinal plants, such as their immunomodulatory, anti-inflammatory, antioxidant, and skin-protective properties, have garnered growing interest [1]. Among these, plant-derived glucomannans constitute a prominent class of β-linked heteropolysaccharides comprising primarily mannose and glucose residues [2]. Their physicochemical profiles and biological potencies are inherently determined by molecular weight distribution, monosaccharide profile, glycosidic bond types, and subtle chemical modifications (particularly acetylation), all of which collectively shape their chain conformations and functional performance [3].

*Bletilla striata*, a traditional medicinal plant extensively utilized in East Asia, has long been for wound healing, anti-inflammatory properties, as well as its applications in functional foods [4] and skin care products [5]. Its tubers are rich in glucomannan-type polysaccharides, which are considered the primary bioactive components responsible for its therapeutic efficacy [6]. Although recent studies have preliminarily characterized their monosaccharide composition and molecular weight distribution [7, 8], research focusing on the comprehensive characterization and detailed structural elucidation of *B. striata* glucomannan remains limited.

The biological activities of polysaccharides extracted from *Bletilla striata* have been extensively documented, with prior studies highlighting their significant pharmacological potential, including excellent biocompatibility, robust antioxidant capacity, and potent immunomodulatory functions [9]. These findings underscore their promising applications in functional materials and biomedical fields.

However, in terms of their structural characterization, most existing research has focused primarily on crude extracts or partially characterized fractions, leaving their fine chemical structures largely undefined. As a result, the underlying structure–property relationships remain unclear, particularly how their molecular architecture correlates with rheological behavior and biological performance. Specifically, the influence of backbone architecture, mannose/glucose distribution, and partial *O*-acetyl substitution on the viscoelastic behavior, intermolecular association, and antioxidant activity of *B. striata* glucomannan has not been fully elucidated. A comprehensive structural characterization combined with functional evaluation is therefore essential to better understanding the molecular basis of its performance and facilitating its rational application in biomedical and cosmetic formulations.

In the present study, a homogeneous glucomannan (labeled as BSP60) was isolated from *B. striata* tubers using a purification regime involving hot-water extraction, ethanol precipitation and subsequent fractionation via size-exclusion chromatography. After confirming its molecular weight and purity via HPSEC-RID, the detailed chemical structure of BSP60 was characterized using monosaccharide composition analysis, methylation analysis, and comprehensive 1D and 2D NMR spectroscopy. In addition, the rheological properties of BSP60 were compared with those of dextran of similar molecular weight to assess the impact of molecular structure on viscoelastic behavior [10]. Furthermore, the cytocompatibility, anti-inflammatory activity, and free radical scavenging capacity of BSP60 were evaluated to explore its potential as a multifunctional natural polysaccharide for biomedical and cosmetic applications. By correlating fine structural characteristics with rheological performance and bioactivities, this work provides new insights into the molecular determinants governing the functionality of *B. striata* glucomannan. These findings establish a clearer structure–function framework and support the application of structurally defined polysaccharides as multifunctional biomaterials in biomedical and skin-related formulations.

## Materials and methods

### Materials

Tianjin Damao Chemical Reagent Factory (Tianjin, China) provided sodium hydroxide, absolute ethanol, sodium chloride, silver nitrate, glacial acetic acid, *n*-butanol, and potassium dihydrogen phosphate (KH_2_PO_4_). The concentrated sulfuric acid and hydrochloric acid utilized in this research were respectively procured from Sichuan Xilong Scientific Co., Ltd (Sichuan, China) and Chongqing Chuandong Chemical Co., Ltd (Chongqing, China). Macklin (Shanghai, China) served as the provider for phenol. Dextran molecular weight standards and sodium borodeuteride (NaBD_4_) were obtained from the National Institutes for Food and Drug Control (Beijing, China) and J&K Scientific Inc. (Guangdong, China), respectively. Sigma-Aldrich (Shanghai, China) supplied the high-purity monosaccharide standards, consisting of d-glucose (Glc) and d-mannose (Man). In addition, reagents essential for the hydrolysis and derivatization processes, namely dimethyl sulfoxide (DMSO), 1-phenyl-3-methyl-5-pyrazolone (PMP), and trifluoroacetic acid (TFA), were sourced from Aladdin (Shanghai, China). Methanol and acetonitrile were from Mreda Technology Inc. (China). Iodomethane and pyridine were from Adamas Reagent Co., Ltd (China). Chloroform and acetic anhydride were from Rianlon Bohua Pharmaceutical Chemical Co., Ltd (China). Deuterium oxide (D_2_O) was from Cambridge Isotope Laboratories, Inc (Cambridge, USA). Tubers of *Bletilla striata* were obtained from Yulin Forestry Development Co., Ltd (Pu’er, China). Acetonitrile was of HPLC grade, unless otherwise indicated, all other reagents were of analytical purity.

### Extraction and purification of the polysaccharides

Initially, 200 g of powdered and dried *B. striata* tubers were immersed in 2.0 L of deionized water and heated at 80 °C for 2 h. To separate the supernatant, the solution was centrifuged at 4000×*g* for 20 min. Subsequently, the remaining solids underwent a second extraction using identical parameters, after which the supernatants from both rounds were pooled. To eliminate starch, α-amylase was introduced into the mixture to reach a 0.1% (*v*/*v*) concentration. This solution was then incubated at 70 °C for about 1.5 h until a negative starch test was achieved. Centrifuge (4000×*g*, 20 min) to remove insoluble. To the supernatant, add 95% ethanol to reach 40% (*v*/*v*) alcohol, then centrifuge (4000×*g*, 20 min) to obtain the 40% ethanol-precipitated fraction and its supernatant. To the 40% supernatant, add 95% ethanol with stirring to reach 60% (*v*/*v*), centrifuge (4000×*g*, 20 min) to obtain the 60% precipitate and supernatant. To the resulting 60% supernatant, add 95% ethanol with stirring to reach 80% (*v*/*v*), then centrifuge (4000×*g*, 20 min) to obtain the 80% precipitate. Wash the 40%, 60%, and 80% precipitates once with 95% ethanol and once with absolute ethanol. After centrifugation, re-dissolve the pellets in water and lyophilize to yield crude *Bletilla striata* glucomannan.

To isolate the target fraction, a 400 mg aliquot of crude glucomannan was solubilized in 20 mL of water. The suspension was clarified via centrifugation (4000×*g*, 20 min) and subsequent filtration using a 0.45 μm membrane. The column (Sephadex G-100) was subsequently loaded with the pre-treated solution. Isocratic elution was performed with 0.1 M NaCl, maintaining a steady velocity of 0.1 mL/min while recovering 2 mL fractions. To construct the final elution profile, the total carbohydrate concentration in each fraction was quantified via the phenol–sulfuric acid assay, and the corresponding absorbance values were graphed against the fraction numbers. Pool fractions corresponding to the same elution peak and collect the fraction with the largest peak area. Purification was achieved by employing dialysis tubing with a 500 Da molecular-weight cutoff for desalting, followed by vacuum concentration and lyophilization, thereby obtaining the purified *B. striata* glucomannan. The final purified polysaccharides from *B. striata* was designated as BSP60, respectively. Specifically, the fraction precipitated at a final ethanol concentration of 60% (*v*/*v*) was collected.

### Measurement of molecular weight

The macromolecular dimensions and molecular mass distribution of the purified *B. striata* glucomannan were resolved through HPSEC-RID analysis. By utilizing a refractive index detector (RID) coupled with high-performance size-exclusion chromatography, we achieved an accurate assessment of the polymer’s polydispersity index (PDI) and its overall compositional uniformity [11]. Chromatographic separation was carried out on an Agilent 1260 Infinity system, which comprised a Shodex OHpak SB-804 HQ column (8 mm × 300 mm) and an in-line refractive index (RI) detector. For the mobile phase, a 0.1 M NaCl solution was pumped at a steady flow rate of 0.5 mL/min, while the column temperature was strictly controlled at 35 °C throughout the process. Both the glucomannan samples and dextran standards were prepared at a concentration of 2 mg/mL in the mobile phase, followed by filtration through 0.20 μm membranes and a 30 μL injection. A linear calibration curve was established by correlating the log(*M*_*w*_) of dextran standards (spanning 2.7–300.6 kDa) with their respective elution volumes. The fundamental molecular parameters, including *M*_*w*_, *M*_*n*_, and the resulting dispersity (*M*_*w*_/*M*_*n*_), were determined using the Agilent GPC workstation (v3.4). The presence of a singular, Gaussian-like elution peak was utilized to confirm the macromolecular homogeneity of the purified fraction.

### Structural validation via FT-IR spectroscopy

For structural characterization, a 1.0 mg aliquot of the sample was homogenized with anhydrous KBr powder. The resulting mixture was then compressed into a translucent disc for analysis. Infrared profiles were recorded in the wavenumber range of 400–4000 cm^−1^ using a Bruker Tensor 27 spectrophotometer [12].

### Elucidation of constituent monosaccharides

For the determination of monosaccharide compositions, the sample underwent acid-mediated liberation using 4 M trifluoroacetic acid. Specifically, 1 mL of the solution (sample or standard) was hermetically sealed with an equal volume of TFA and maintained at 110 °C for a 4 h duration. This process preceded the PMP-labeling procedure used for subsequent quantification. Following the complete removal of TFA via rotary evaporation, the dried residue was reconstituted in deionized water. The derivatization reaction was initiated by the addition of a reagent mixture (0.6 M NaOH and 0.5 M PMP in methanol), followed by allowing the mixture to react at 70 °C for 60 min. Once the solution reached room temperature, it was neutralized using 0.6 M HCl. The unreacted PMP was then extracted three times with 2 mL of chloroform. After recovering the aqueous phase, it was subjected to centrifugation (15,000×*g*, 10 min) and filtered through a 0.22-μm membrane to eliminate any solid particles. The subsequent analysis was performed on an Agilent 1260 HPLC apparatus fitted with a ZORBAX SB-C18 column (250 mm × 4.6 mm, 5 μm) and a diode array detector (DAD). The column oven was set to 25 °C. For the separation, a solvent system comprising phase A (acetonitrile/pH 6.9 KH_2_PO_4_ buffer, 15:85, *v*/*v*) and phase B (the identical solvents in a 40:60, *v*/*v* ratio) was utilized. The elution was conducted over a 60-min gradient at 1.0 mL/min, with a final 5-min re-equilibration using only phase A. Chromatographic analysis was performed with an injection volume of 10 μL and UV detection at 250 nm. Monosaccharide standards were processed identically to samples to enable peak identification and quantitation [13].

### Methylation and GC–MS analysis

The linkage configurations were resolved through a PMAA strategy utilizing a refined DMSO/NaOH-methyl iodide protocol [14]. The methylation-initiating medium was prepared by homogenizing 50 mg of anhydrous NaOH into 1 mL of anhydrous DMSO. An aliquot of the polysaccharide (3–5 mg) was then solubilized in this caustic reagent, followed by a cryogenic treatment to facilitate the formation of a solid matrix. After the immediate addition of 1 mL CH_3_I, the sealed vessel was subjected to sonication at ambient temperature for 1 h in total darkness. The reaction was ended with 2 mL water, and the methylated products were recovered through three successive extractions with chloroform (1:1, *v*/*v*). The pooled organic phases were rinsed once with deionized water and subsequently concentrated via rotary evaporation under reduced pressure. For the liberation of partially methylated monosaccharides, the resulting residue was subjected to acid hydrolysis using 2 mL of 2 M trifluoroacetic acid (TFA). This reaction was carried out in a hermetically sealed tube at 120 °C for a duration of 2 h [14]. The pH of the solution was adjusted to 10 with NaOH (1 M) and reduced with NaBD_4_ (25 mg) at 50 °C for 2 h; the reaction was terminated by adding 100 μL glacial acetic acid. Solvents were removed by rotary evaporation, and borates were eliminated by successive additions of 10% methanolic acetic acid followed by methanol. The dried residue underwent peracetylation by reacting with a 1:1 mixture of pyridine and acetic anhydride (0.5 mL each) within a sealed, pressure-resistant vial. This thermal conversion was maintained at 100 °C for 1 h. To neutralize excess reagents, 1 mL of deionized water was introduced, followed by the selective extraction of the resulting PMAA derivatives into methylene chloride (3 × 2 mL). The pooled organic extracts were rinsed twice with equal volumes of water, concentrated to a residual volume of approximately 100 μL, and subsequently reconstituted in 500–600 μL of chloroform. Following filtration through a membrane (0.22 μm), the final solution was ready for GC–MS characterization.

The structural characterization of the partially methylated alditol acetate (PMAA) derivatives was performed on a gas chromatograph-mass spectrometer (GC–MS), following the specific method previously reported by our team [2].

### NMR spectroscopy analysis

To NMR characterization, 20 mg of *B. striata* glucomannan was dissolved in 1 mL of D_2_O, subsequently frozen and lyophilized, and the deuterium exchange was repeated on three occasions. The resulting dry material was reconstituted in 0.5 mL of D_2_O containing 0.05 wt% TMSP-*d*_4_ (sodium 3- (trimethylsilyl)propionate-2,2,3,3-*d*₄) as the chemical-shift reference (*δ*_H_/*δ*_C_ = 0.00) [15]. One- and two-dimensional NMR spectra were obtained on a Bruker Avance 800 MHz spectrometer equipped with a ^1^H/^13^C probe operating, including one-dimensional experiments for ^1^H and ^13^C, and coherence-related experiments (COSY, TOCSY, ROESY), and heteronuclear ^1^H–^13^C HMBC experiments (Heteronuclear Multiple-Quantum Correlation). Spectral processing and assignment were performed with the software program MestReNova.

### Rheological properties

Steady-state rheological measurements were executed on an MCR 302e rheometer (Anton Paar). A parallel-plate configuration (diameter: 25 mm) was selected as the sensing system, with the sample thickness maintained at a constant 1 mm gap to ensure uniform shear distribution [16].

#### Steady-state shear test

To obtain steady-state flow curves, 10 mg/mL aqueous solutions of BSP60 and dextran were prepared. Measurements were performed in flow sweep mode. The temperature was maintained at 25 °C with a shear-rate sweep range of 0.01–1000 s^−1^.

#### Dynamic rheological test

Frequency-dependent moduli were recorded within the linear viscoelastic limits of the samples, as identified by preliminary strain sweeps. A prescribed strain amplitude of 1% was utilized across a frequency spectrum from 0.1 to 300 rad/s. All oscillatory measurements were performed at a stabilized temperature of 25 °C, facilitated by a Peltier thermal regulator (±0.1 °C) and a 25 mm parallel-plate configuration (gap: 1 mm).

### Cytocompatibility assay

To evaluate the cytocompatibility of the extract, RAW 264.7 cells were seeded (10000 cells per well) and allowed to equilibrate overnight in 96-well plates. After a 24 h exposure to *B. striata* extract at concentrations of 0.1, 0.3, and 0.5%, colorimetric assessment was initiated by introducing 0.5 mg/mL MTT. The formation of formazan crystals was allowed to proceed for 4 h at 37 °C before further analysis. Upon completion, the culture medium was discarded, and the resulting formazan precipitates were solubilized by the addition of 150 µL DMSO [17]. The optical density was quantified at a primary wavelength of 490 nm, utilizing 570 nm as the background reference. The resulting spectrophotometric data were subsequently imported into Origin software (Version 2021) for comprehensive statistical evaluation and graphical representation.

### Determination of inflammatory mediators

RAW 264.7 cells (2 × 10^5^ cells/well) were first equilibrated overnight. At 40–60% confluency, the medium was supplemented with *B. striata* extract (0.3%). Following this protective pre-treatment for 2 h, the cells were challenged with 1 μg/mL LPS. The incubation period was extended for another 22 h to allow for the expression of inflammatory mediators [18]. Post-incubation, the supernatants from the cell cultures were harvested and preserved at −80 °C for subsequent analysis. To quantify the concentrations of NO alongside the cytokines IL-6 and TNF-α, commercially available assay kits supplied by Beyotime Biotechnology (Shanghai, China) were employed. All procedures were performed according to the manufacturer’s instructions. Ultimately, a microplate reader was utilized to record the absorbance values, which were read at 450 nm for IL-6 and TNF-α, and at 540 nm for the NO assay.

### Free radical scavenging activity

#### ABTS^+•^ scavenging assay

ABTS^+•^ radicals were generated by mixing ABTS solution (4.06 mg/mL) with ammonium persulfate solution (0.593 mg/mL) at an equal volume ratio, followed by incubation in the dark at 4 °C for 12 h. The resulting reaction mixture was subsequently diluted 30-fold with phosphate-buffered saline (PBS) to obtain the ABTS^+•^ working solution [19].

For the assay, 80 μL of BSP60 solution was added to a 96-well microplate as the test group (As). A hyaluronic acid (HA) solution at the same concentration was utilized as a reference control, while 80 μL of phosphate-buffered saline (PBS) served as the negative control (Ac). A volume of 120 μL ABTS⁺^•^ working solution was dispensed into every well. The reaction mixture was incubated in the dark at ambient temperature for 30 min, then determined at 734 nm with a microplate reader (FlexStation 3 Multi-Mode Microplate Reader, USA).

The ABTS^+•^ scavenging activity was calculated as follows:

$${\mathrm{ABTS}}^{ + \bullet } {\mathrm{scavenging}}\,{\mathrm{ratio}}(\% ) = \frac{{A_{c} - A_{s} }}{{A_{c} }} \times 100\%$$

#### O_2_^•−^ free radical scavenging assay

To formulate the O_2_^•−^ (superoxide anion radical) generating system, a mixed solution was prepared by sequentially combining 10 mL of methionine (11.9 mg/mL), 25 mL of nitroblue tetrazolium (NBT, 0.156 mg/mL), and 5 mL of riboflavin (94 μg/mL). Subsequently, 10 mL of phosphate buffer solution (PBS) was introduced into the mixture, followed by thorough homogenization. To prevent premature degradation, this freshly prepared working reagent was strictly protected from light until immediately required for the assay.

For the measurement, 120 μL of BSP60 solution was added to a 96-well plate as the experimental group (As). Hyaluronic acid (HA) solution at the same concentration was included as a comparative control, and 120 μL of PBS was used as the negative control group (Ac). Subsequently, 100 μL of the O_2_^•−^ working solution was added to each well. The plate was exposed to LED illumination, the light intensity of which was constant, for a period of 5 min in order to initiate the reaction. Subsequently, the optical density (OD) was quantified at a detection wavelength of 560 nm utilizing a Multiskan FC microplate spectrophotometer (Thermo Scientific, USA) [20].

The O_2_^•−^ scavenging efficiency was calculated as follows:

$${\mathrm{O}}_{2}^{ \bullet - } {\mathrm{scavenging}}\,{\mathrm{ratio}}(\% ) = \frac{{{\mathrm{A}}_{{\mathrm{c}}} - {\mathrm{A}}_{{\mathrm{s}}} }}{{{\mathrm{A}}_{{\mathrm{c}}} }} \times 100{{\% }}$$

#### Statistical analysis

To ensure reproducibility, each experimental procedure was replicated a minimum of three independent times. The accumulated data are presented as the mean accompanied by the standard deviation (±SD). For the evaluation of statistical significance between distinct groups, an independent samples Student’s *t* test was executed. The threshold for statistical significance was established at *p* < 0.05. Furthermore, Microsoft Excel (Microsoft Corp., Redmond, WA, USA) served as the primary tool for data processing, while all graphical representations were rendered using Origin 2021 software.

## Results and discussion

### Extraction and purification of BSP60

The crude polysaccharide of *B. striata* was isolated via hot water extraction followed by gradient ethanol precipitation. Specifically, 53.56 g of crude polysaccharide was obtained from 200.00 g of raw materials, corresponding to an extraction yield of 26.78%. The purification of 400 mg of crude polysaccharide using Sephadex G-100 size-exclusion chromatography yielded 350 mg of a refined polysaccharide fraction, designated as BSP60, with a yield of 87.5% relative to the crude extract. The integrity and homogeneity of the sample were verified by high-performance gel permeation chromatography (HPGPC), which produced a sharp, unimodal peak without significant baseline drift. This chromatographic profile confirms that the obtained polysaccharide possesses a highly uniform molecular weight distribution, indicative of its high purity.

### Molecular weight and molecular weight distribution of BSP60

**Fig. 1:**
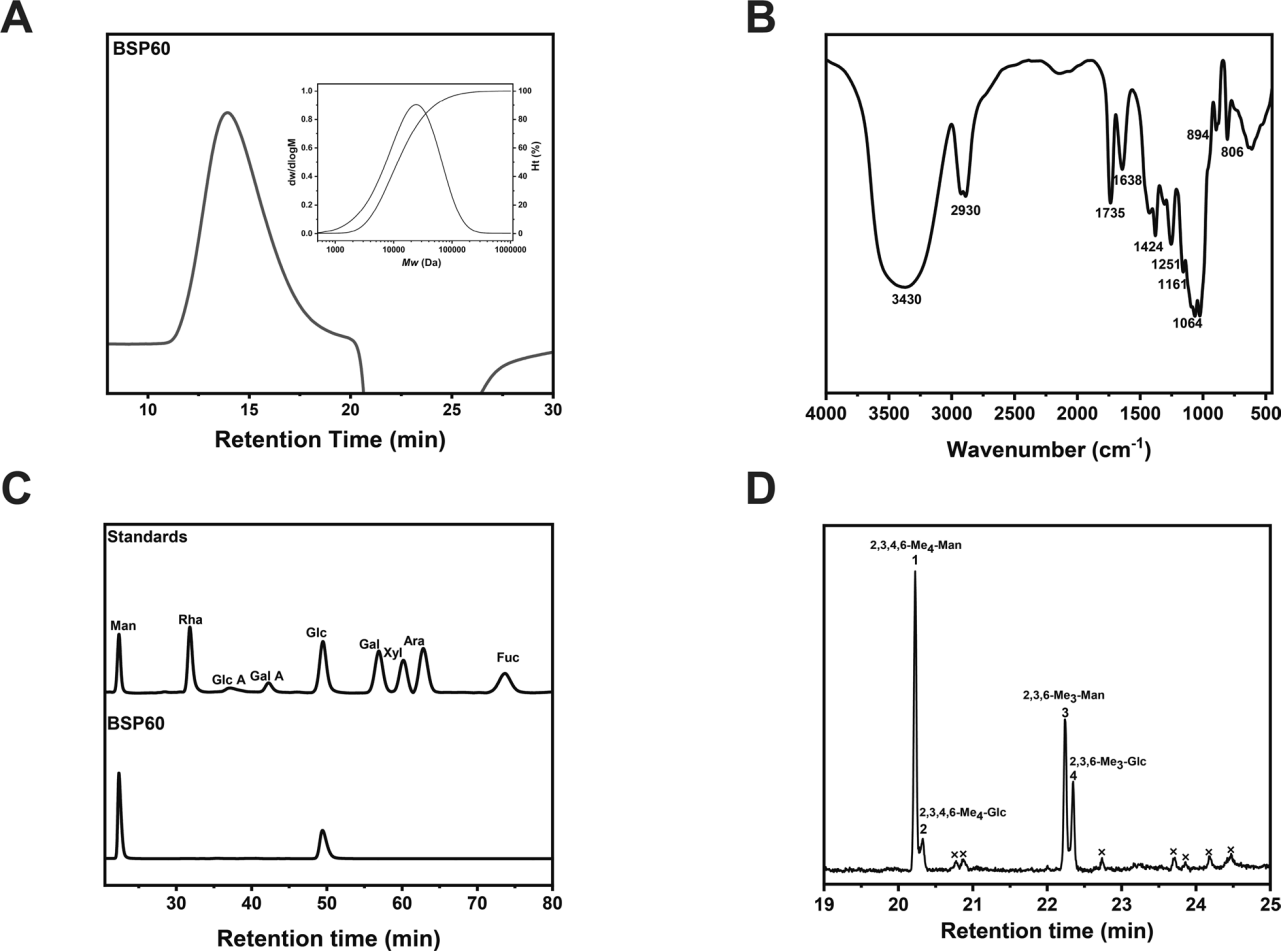
The characterization data for BSP60 **A** Elution profiles reflecting molecular weight distributions; **B** FT-IR vibrational spectra; **C** HPLC chromatograms of the PMP-derivatized sample and reference monosaccharides; **D** the total ion chromatogram (TIC) profiles of partially methylated alditol acetates (PMAAs) from the polysaccharides

As shown in Fig. 1A, GPC software analysis determined that the weight-average molecular weight (*M*_*w*_) of the sample was determined to be 30,609 Da, with a number-average molecular weight (*M*_*n*_) of ∼10,591 Da. Accordingly, the calculated polydispersity index (PDI) was 2.89. While this PDI value reflects a typical polydisperse distribution characteristic of natural plant polysaccharides, the unimodal HPGPC profile indicates that the purified sample represents a homogeneous, successfully isolated polysaccharide fraction.

### FT-IR spectra analysis of BSP60

In Fig. 1B, the FT-IR spectrum of BSP60 exhibits a broad, intense absorption band centered at approximately 3430 cm^−1^, which is characteristic of hydrogen-bonded O–H stretching vibrations in polysaccharides [21]. A weaker absorption at ~2930 cm^−1^ originates from aliphatic C–H stretching of ring methine/methylene groups [22]. In the carbonyl region, a discernible band at ~1735 cm^−1^ [21], accompanied by an additional band at ~1251 cm^−1^, can be assigned to the C=O and C–O stretching vibrations of *O*-acetyl ester substituents, respectively, indicating the presence of partial acetylation. The absorption signal near ~1638 cm^−1^ is primarily attributable to the H–O–H bending of bound/adsorbed water (with possible minor overlap from carboxylate). A band at ~1424 cm^−1^ corresponds to CH/CH_2_ bending vibrations, including the deformation vibration of the acetyl CH₃ deformation [23]. In the fingerprint region, strong absorptions at ~1161 and ~1064 cm^−1^ are typical of C–O–C/C–O stretching and pyranose ring skeletal vibrations. A prominent band at ~894 cm^−1^ is diagnostic of β-type glycosidic linkages, while the feature at ~806 cm^−1^ arises from pyranose ring skeletal and out-of-plane vibrations. Taken together, these spectral features indicate that BSP60 could be a β-glucan, i.e., a polysaccharide composed predominantly of β-linked glucopyranose residues with minor *O*-acetyl substituents.

### Monosaccharide composition analysis of BSP60

As shown in Fig. 1C, the sample chromatogram was compared with a mixture of authentic mannose and glucose standards. Comparative analysis of retention times confirmed that BSP60 is primarily constituted of glucose and mannose. Molar ratios calculated from peak areas further validated the dominant contribution of these two hexoses to the carbohydrate structure. After pre-column derivatization with PMP, HPLC analysis of BSP60 yielded two distinct peaks corresponding to mannose and glucose, with a molar ratio of approximately 7:3, confirming that the BSP60 consists exclusively of these two monosaccharides.

### Methylation and GC–MS analysis of BSP60

Based on the GC–EIMS spectral library of the Complex Carbohydrate Research Center (CCRC, University of Georgia), combined with the corresponding fragmentation patterns and retention characteristics (Table 1; Fig. 1D), four distinct PMAA derivatives were identified in the BSP60 sample.

**Table 1:** GC–MS data for the linkage positions of monomeric units in BSP60

Retention time (min)	Molar ratio	Mass fragments (*m*/*z*)	Type of linkages
20.226	13.45	71, 87, 101, 117, 129, 145, 161, 205	2,3,4,6-Me_4_-Man
22.246	7.48	71, 87, 101, 117, 129, 143, 161, 233	2,3,6-Me_3_-Man
20.329	1.00	71, 87, 101, 117, 129, 145, 161, 205	2,3,4,6-Me_4_-Glc
22.349	3.66	71, 87, 99, 117, 131, 142, 161	2,3,6-Me_3_-Glc

The methylated derivatives, along with their corresponding retention times and characteristic mass fragments, are summarized in Table 1.

These results collectively demonstrate that BSP60 possesses a backbone composed of (1 → 4)-linked mannopyranosyl and (1 → 4)-linked glucopyranosyl residues, with chain termination by non-reducing terminal mannopyranosyl and glucopyranosyl residues.

Molar ratio analysis (Table 1) revealed that terminal mannose (13.45) was the most abundant residue, followed by (1 → 4)-linked mannose (7.48), (1 → 4)-linked glucose (3.66), and terminal glucose (1.00). The disproportionately high molar ratio of terminal mannose residues (13.45) relative to (1 → 4)-linked backbone residues (7.48) is attributed to partial alkaline hydrolysis of the polysaccharide chains during the methylation process [24].

### NMR spectra analysis

Comprehensive NMR spectroscopy was employed to elucidate the fine structure of BSP60. Specifically, anomeric configurations were determined by analyzing chemical shifts and coupling constants; individual sugar residues were mapped using COSY and TOCSY; and glycosidic linkages and sequence arrangements were confirmed via HMBC and NOESY experiments [25].

**Fig. 2:**
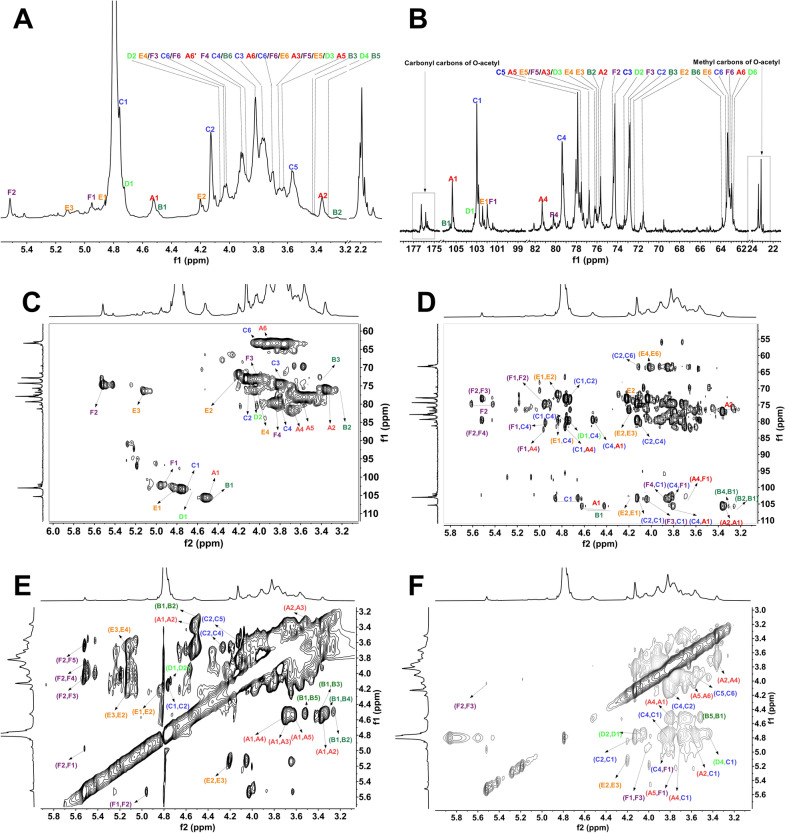
The NMR spectra of BSP60. **A** ^1^H-NMR of BSP60; **B** ^13^C-NMR of BSP60; **C** ^1^H–^13^C HSQC of BSP60; **D** ^1^H–^13^C HMBC of BSP60 **E** ^1^H–^1^H TOCSY of BSP60. The **A** (red), B (green), C (blue), D (neon green), E (orange) and F (purple) represent → 4)-β-d-Glc-(1 → , β-d-Glc-(1 → 4), → 4)-β-d-Man-(1 → , β-d-Man-(1 → 4), → 4)-3-*O*-acetyl-β-d-Man-(1 → and → 4)-2-*O*-acetyl-β-d-Man-(1 → , respectively

Comprehensive NMR datasets (^1^H, ^13^C, and 2D correlations) were acquired to resolve the linkage patterns of BSP60. The ^1^H NMR and ^13^C NMR spectra of *B. striata* glucomannan have been demonstrated in Fig. 2. All the ^1^H- and ^13^C-NMR chemical shifts of BSP60 are presented in Table 2. Signal integration of the ^1^H NMR spectrum (Fig. 2A) revealed an approximate Man:Glc molar ratio of 2.3:1, calculated from the relative integrated intensities of six distinct residues (A–F) with an ratio of 2.2:0.9:4.2:1.0:1.0:1.1. The anomeric proton signals defining these residues appeared at *δ*_H_ 4.52, 4.50, 4.76, 4.73, 4.84, and 4.96 ppm, corresponding to → 4)-β-d-Glcp-(1 → (A), terminal → 4)-β-d-Glcp-(1 → (B), → 4)-β-d-Manp-(1 → (C), terminal → 4)-β-d-Manp-(1 → (D), 3-*O*-acetyl- → 4)-β-d-Manp-(1 → (E), and 2-*O*-acetyl- → 4)-β-d-Manp-(1 → (F), respectively. Additionally, methyl proton signals from the acetylated groups were observed in the region of *δ*_H_ 2.10–2.30 ppm. Residues E and F represent mannose units acetylated at O-3 and O-2, respectively, collectively accounting for about 23.54% of total monosaccharide residues on a molar basis [2].

**Table 2:** ^1^H- and ^13^C-NMR chemical shifts of polysaccharides BSP60

Monosaccharide units	Chemical shifts (ppm)					
	H1/H1′/C1	H2/C2	H3/C3	H4/C4	H5/C5	H6/H6′/C6
A: → 4)-β-d-Glc-(1 →	4.52 105.4	3.36 75.9	3.66 76.8	3.70 81.4	3.62 77.9	3.76/3.93 63.3
B: β-d-Glc-(1 → 4) (non-reducing end)	4.50 105.2	3.26 75.9	3.41 72.4	– 75.8	– –	3.83 63.1
C: → 4)-β-d-Man-(1 →	4.76 103.0	4.13 72.9	3.79 74.4	3.82 79.6	3.56 78.0	4.03 63.3
D: β-d-Man-(1 → 4) (non-reducing end)	4.73 103.2	4.10 73.1	3.70 76.8	3.41 72.4	– –	– –
E: → 4)-3-*O*-acetyl-β-d-Man-(1 →	4.84 102.8	4.20 71.5	5.12 76.2	4.04 75.9	3.66 77.0	3.88 63.3
F: → 4)-2-*O*-acetyl-β-d-Man-(1 →	4.96 102.0	5.52 74.4	4.03 73.2	3.88 79.4	3.66 77.0	3.75 63.3

*ND* not determined

In the ^13^C NMR spectrum (Fig. 2B), anomeric carbon signals were detected at *δ*_C_ 100–106 ppm, while acetyl carbonyl carbons appeared at *δ*_C_ 175–177 ppm, and acetyl methyl carbons around *δ*_C_ 23.0 ppm. The C-4 signals of residues A and C were observed at *δ*_C_ 81.3 and 79.4 ppm [26], respectively, with corresponding proton resonances at *δ*_H_ 3.70 and 3.82 ppm; both signals were downfield-shifted relative to unsubstituted Glc and Man, indicating substitution at the O-4 positions. For residue F, the proton signals of H-2 and H-4 were observed at δ_H_ 5.51 and 4.04 ppm, with corresponding carbon signals at *δ*_C_ 74.5 and 75.9 ppm, respectively, both downfield-shifted compared with unsubstituted mannose, confirming *O*-acetyl substitution at C-2 and glycosidic linkage at C-4 [27]. Similarly, residue E exhibited H-3 and H-4 resonances at *δ*_H_ 5.08 and 4.04 ppm, with C-3 and C-4 signals at *δ*_C_ 71.5 and 75.7 ppm, respectively, confirming acetylation at O-3 and glycosidic linkage at O-4. Residues E and F are mannose units acetylated at O-3 and O-2, respectively, accounting for about 23.54% of the total monosaccharide residues on a molar basis.

The non-reducing terminal residue B and residue D exhibited C-1 signals at *δ*_C_ 105.3 and 103.2 ppm and C-4 signals at *δ*_C_ 75.8 and 72.4 ppm [28], respectively. Compared with substituted residues, their C-1 shifts moved downfield, while C-4 shifts moved upfield, indicating their terminal positions. The acetyl groups were characterized by three distinct signals: methyl protons at *δ*_H_ 2.20 ppm, carbonyl carbons at* δ*_C_ 176.3 ppm, and methyl carbons at approximately *δ*_C_ 23.0 ppm. The heteronuclear multiple bond correlation (HMBC) NMR spectrum (Fig. 2D) revealed key long-range heteronuclear correlation signals between anomeric protons and glycosidic bond carbons. A significant correlation peak was observed between the H-1 signal of residue A and the C-4 signal of residue C, confirming the presence of an α-(1 → 4) glycosidic linkage between these two residues. Additional critical correlations included: the H-1 signal of residue C correlated with the C-4 of residue A; the H-1 signal of residue D correlated with the C-4 of residue C; the H-1 signal of residue E correlated with the C-4 of residue C; the H-1 signal of residue F correlated with the C-4 of residue A.

The spatial connectivity of BSP60 was further resolved through ROESY cross-peaks (Fig. 2F). The close proximity of H-1 from residues A, C, D, and F to the H-4 protons of adjacent units (specifically C and A) substantiated the backbone. These results collectively confirmed the (1 → 4) glycosidic scaffolding among the identified monosaccharide moieties.

**Fig. 3:**
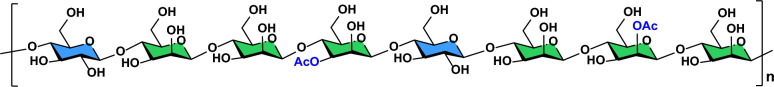
Proposed structure of BSP60

Integration of multidimensional NMR data established that *B. striata* glucomannan features a backbone composed of alternating β-d-1,4-linked glucopyranosyl (A) and mannopyranosyl (C) residues, interspersed with 3-*O*-acetyl- and 2-*O*-acetyl-β-d-1,4-linked mannopyranosyl units (E and F). The polysaccharide chain is terminated with a β-d-Glcp residue (B) at the reducing end and a β-d-mannopyranosyl residue (D) at the non-reducing end. These findings identify BSP60 as a novel polysaccharide, whose proposed structure of which is depicted in Fig. 3.

### Rheological properties

**Fig. 4:**
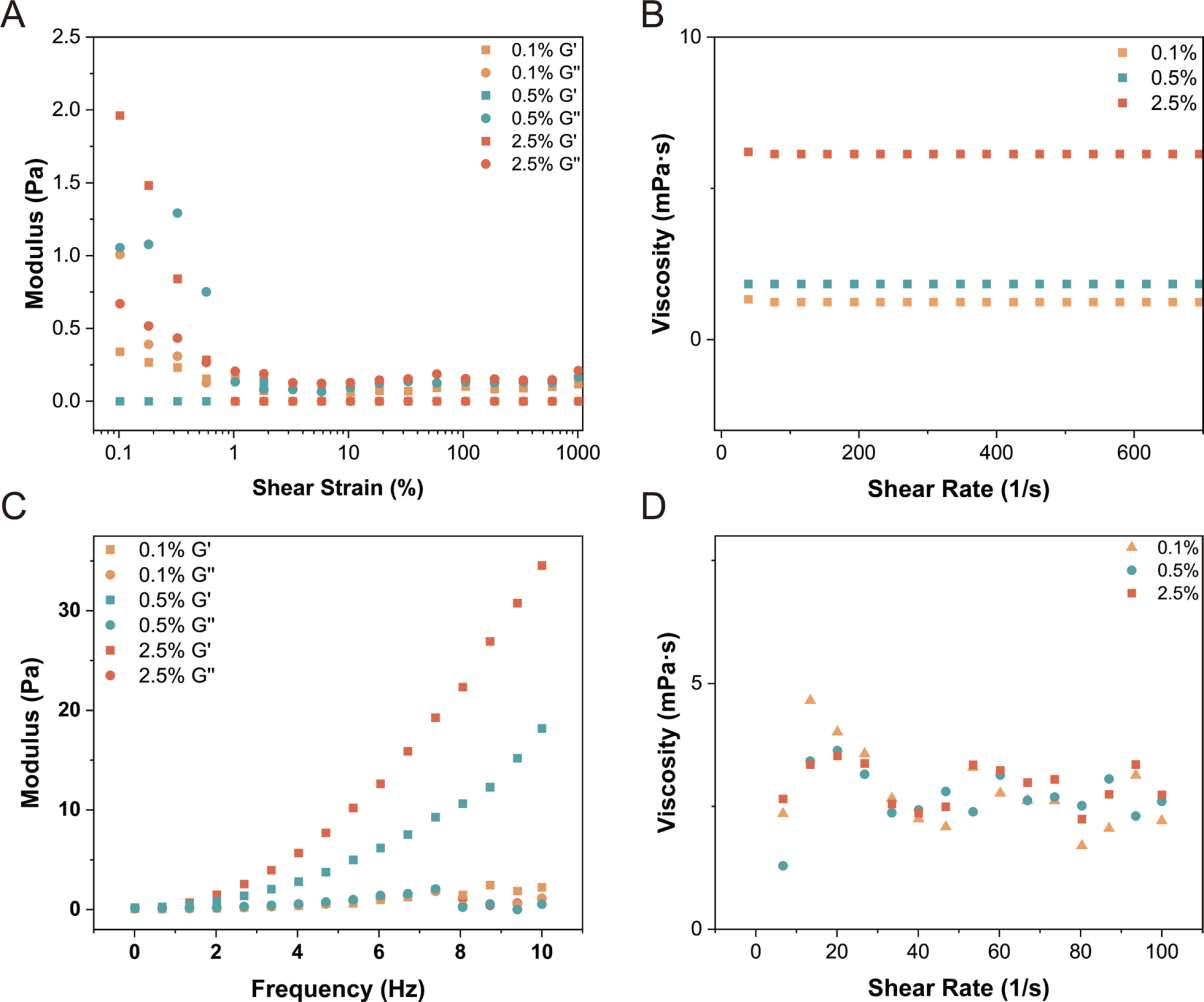
Rheological properties of two polysaccharides at different concentrations: **A** amplitude sweep; **B** apparent viscosity; **C** frequency sweep of BSP60; **D** apparent viscosity of dextran

The rheological profiles of BSP60 and a molecular weight-matched dextran are compared in Fig. 4A–D. Notably, BSP60 Newtonian-like behavior under steady shear, with a relatively constant apparent viscosity throughout the investigated shear rate range. Its apparent viscosity is significantly higher than that of dextran, indicating stronger chain entanglement and intermolecular associations within the BSP60 network. Both storage modulus (*G*′) and loss modulus (*G*″) of BSP60 increase with concentration and frequency, and *G*′ remains consistently higher than *G*″, suggesting that BSP60 forms a viscoelastic network dominated by elastic behavior.

In contrast, dextran exhibits lower viscosity and weaker viscoelasticity, with *G*′ values close to or below *G*″, reflecting its predominantly viscous nature [29]. The distinct rheological difference between BSP60 and dextran can be attributed to the structural influence of mannose residues in BSP60 [30]. It is hypothesized that the cis-oriented hydroxyl groups of mannose residues align on the same side of the sugar ring, facilitating extensive intermolecular hydrogen bonding and consequently contribute to the elevated viscosity [31]. Thus, despite sharing similar molecular weights, the incorporation of mannose significantly enhances the elasticity and structural integrity of BSP60, reflecting stronger intermolecular interactions and more organized network configuration [3]. Collectively, these rheological distinctions suggest that BSP60 holds strong potential as a structure-forming biopolymer for applications requiring enhanced mechanical stability and tunable viscoelasticity, such as biofunctional hydrogels or biomedical matrices. Benefiting from these superior rheological attributes inherent to natural macromolecules, BSP60 demonstrates broad potential in emerging biomedical fields, particularly as pharmaceutical excipients [32], hydrogel scaffolds [33], and microneedle arrays [34].

### Cell compatibility and anti-inflammatory activity

**Fig. 5:**
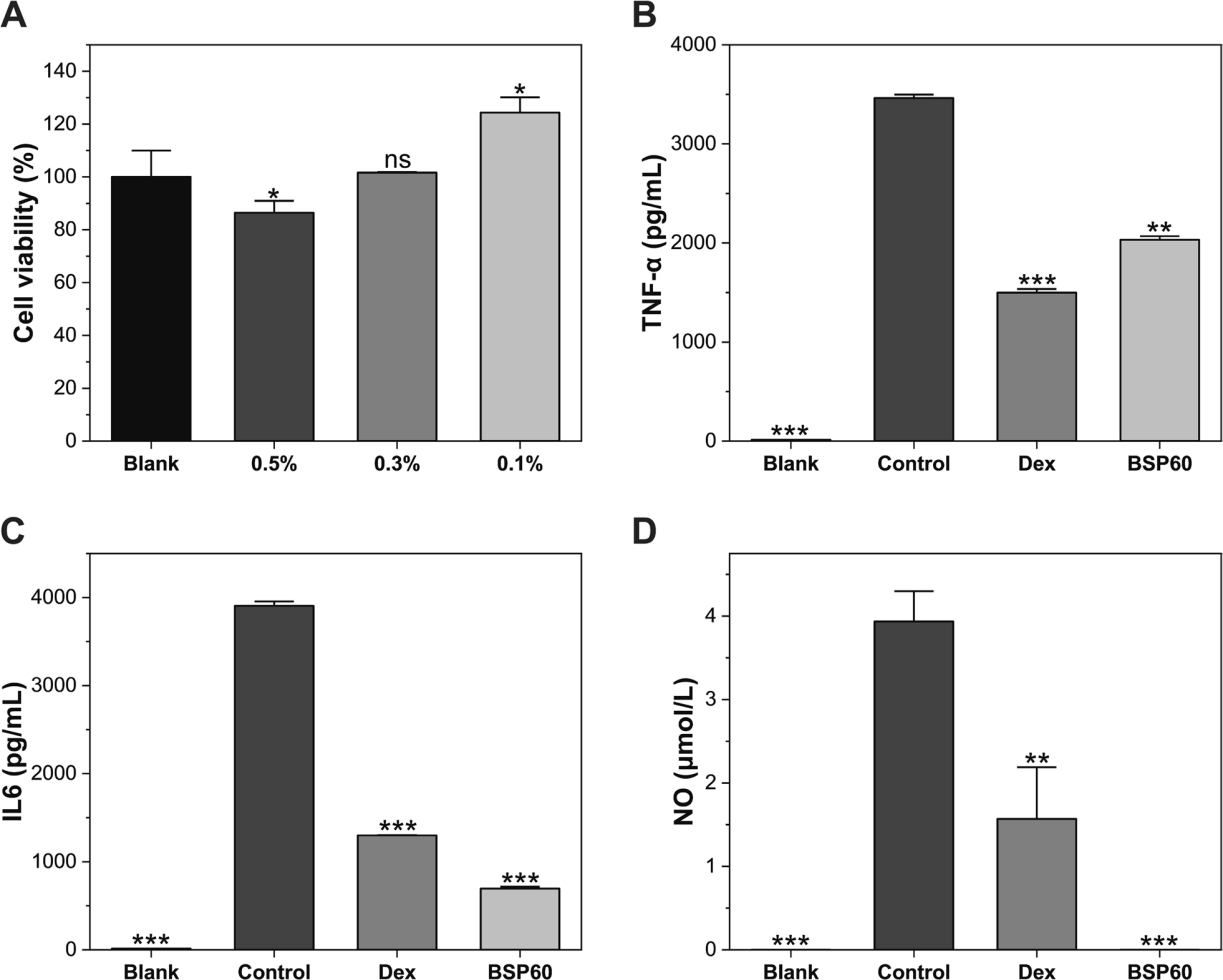
Comparison of cytotoxicity and anti-inflammatory activity. **A** Cytotoxicity of BSP60 at different concentrations (0.1%, 0.3%, and 0.5%) determined by CCK-8 assay. The levels of TNF-α (**B**), IL-6 (**C**), and NO (**D**) secretion in LPS-stimulated cells treated with BSP60 or dexamethasone (Dex). Data points represent the average values ± SD (*n* = 3). Asterisks (*^,^ **^,^ ***) denote significant differences at *p* < 0.05, 0.01, and 0.001, respectively, in comparison with the LPS-treated model

As shown in Fig. 5A, cell viability remained above 100% at concentrations of 0.1% and 0.3%, indicating no detectable cytotoxic effects within this range. Notably, treatment with 0.1% BSP60 enhanced cell proliferation [35], with viability reaching approximately 124%. At a higher concentration of 0.5%, cell viability slightly decreased to about 86%, suggesting a modest inhibitory effect at elevated levels. Collectively, these results indicate that BSP60 exhibits excellent cytocompatibility at concentrations below 0.5%.

The anti-inflammatory effects of BSP60 were evaluated by quantifying the production of pro-inflammatory mediators (TNF-α, IL-6, and NO) in inflammation-induced cells. Comparison between the blank and control groups (Fig. 5B–D) revealed robust upregulation of pro-inflammatory markers. The significant difference in mediator release between untreated cells and the LPS-challenged model confirms that the inflammatory state was successfully induced. For TNF-α, BSP60 treatment reduced secretion levels by approximately 41% compared to the model group (Fig. 5B). In the case of IL-6, the treatment resulted in an over 80% reduction, demonstrating efficacy comparable to that of the positive control, dexamethasone (Fig. 5C). Furthermore, BSP60 significantly decreased the concentration of NO relative to the model group, restoring it to levels similar to the blank group (Fig. 5D). Collectively, these findings demonstrate the potent anti-inflammatory efficacy of BSP60, as evidenced by its strong ability to suppress the secretion of key pro-inflammatory cytokines and mediators [36]. Accordingly, bioactive glycans such as BSP60 hold considerable potential for development as functional cosmetic ingredients [36, 37].

### Antioxidant and radical scavenging activity

**Fig. 6:**
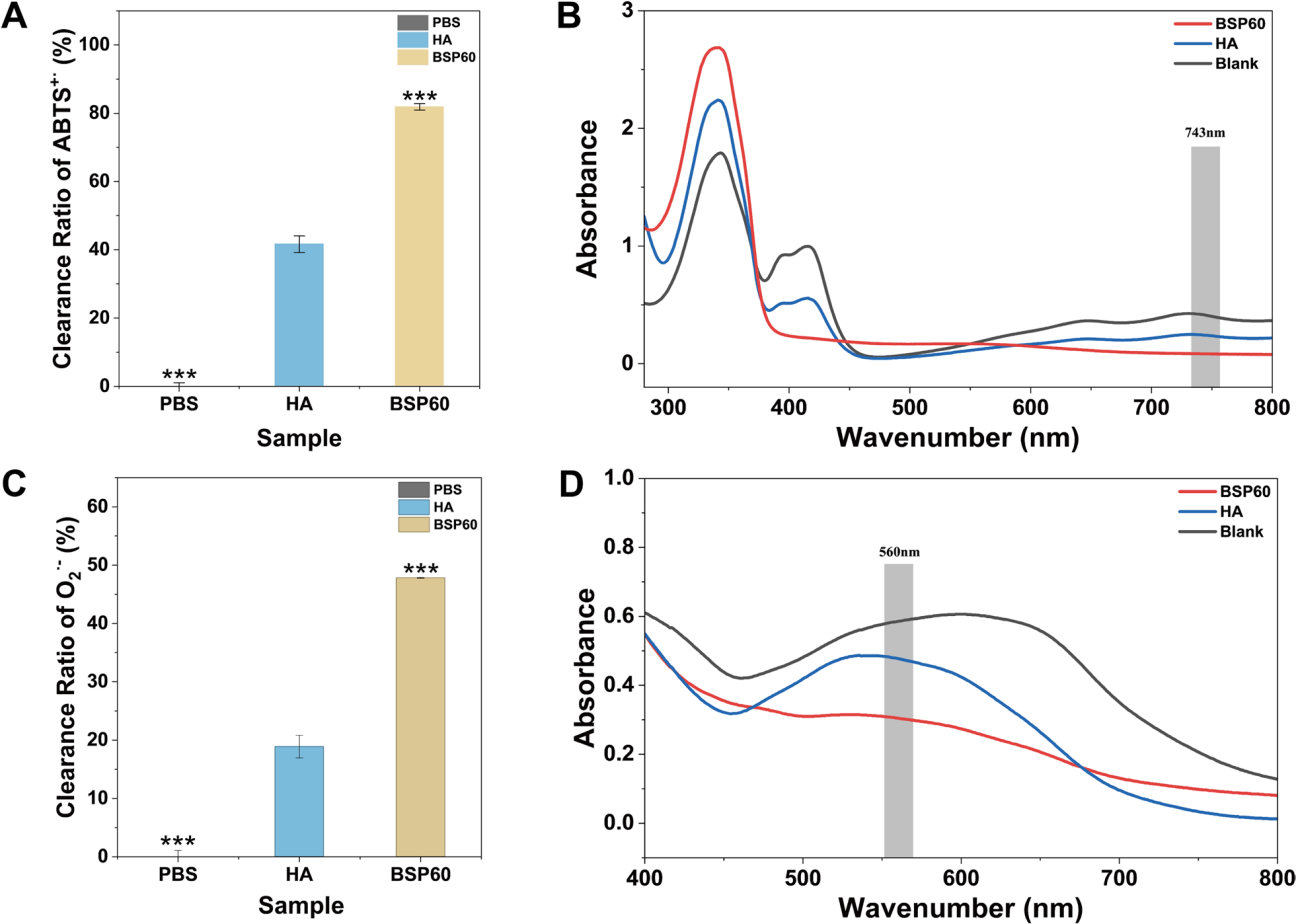
Antioxidant and free radical scavenging activity of BSP60. ABTS⁺^•^ radical scavenging efficiency of BSP60 and HA at 20 mg/mL (**A**) and the corresponding UV–vis absorbance spectra of the reaction solutions (280–800 nm) (**B**). Superoxide anion (O_2_^•−^) scavenging efficiency of BSP60 and HA at 20 mg/mL (**C**) and the corresponding UV–vis absorbance spectra of the reaction solutions (400–800 nm) (**D**). Data are presented as mean ± SD (*n* = 3). Asterisks (*^,^ **^,^***) denote significant differences at *p* < 0.05, 0.01, and 0.001, respectively, in comparison with the LPS-treated model

As shown in Fig. 6, the antioxidant capacity of BSP60 was evaluated using ABTS^+•^ and O_2_^•−^ radical scavenging assays. For comparative consistency, BSP60 and hyaluronic acid (HA) were tested at identical concentrations, with PBS serving as the negative control. In the ABTS^+•^ scavenging assay, BSP60 exhibited a significantly higher radical scavenging efficiency than HA and PBS, achieving an ABTS⁺^•^ scavenging ratio exceeding 80% (Fig. 6A) [38]. In contrast, HA showed only moderate scavenging activity, while PBS displayed negligible activity. Consistent with these quantitative results, UV–vis absorption spectra revealed attenuation of the characteristic ABTS^+•^ absorption peak in the presence of BSP60, confirming its strong electron- or hydrogen-donating capacity toward ABTS^+•^ radicals (Fig. 6B).

A similar trend was observed in the O_2_^•−^ scavenging assay. BSP60 demonstrated substantially higher superoxide radical scavenging efficiency, reaching approximately 50%, which was markedly greater than that observed for HA and the negative control group (Fig. 6C) [5]. Corresponding UV–vis spectra further supported this observation, showing a clear reduction in absorbance associated with NBT reduction in the BSP60-treated group, indicating effective suppression of O_2_^•−^ generation (Fig. 6D). Notably, HA exhibited limited scavenging capacity in this assay system, suggesting its relatively weak activity under identical experimental conditions.

Overall, these results demonstrate that BSP60 possesses significantly stronger free radical scavenging capability than HA when evaluated at the same concentration. The superior antioxidant performance of BSP60 in both ABTS^+•^ and O_2_^•−^ systems suggests that its unique chemical structure may facilitate more efficient interactions with reactive oxygen species [32]. These antioxidant properties provide a plausible mechanistic basis for the protective effects of BSP60 against oxidative stress and support its potential application in skin-protective and bioactive materials [39].

## Conclusion

A glucomannan polysaccharide (BSP60) was isolated from *Bletilla striata* and comprehensively characterized. Methylation GC–MS and multidimensional NMR analyses demonstrated that BSP60 is a β-(1 → 4)-linked glucomannan composed of alternating glucose and mannose residues, with partial *O*-acetylation at C-2 and C-3 positions of mannose units. The high mannose content and moderate acetylation degree confer structural flexibility, which contributes to its unique physicochemical properties. Rheological analysis revealed BSP60 exhibits shear-thinning behavior and forms a predominantly elastic viscoelastic network, with significantly higher viscosity and storage modulus compared to dextran of similar molecular weight. These characteristics suggest enhanced intermolecular associations and the formation of a transient viscoelastic network structure. Biological assays showed that BSP60 has excellent cytocompatibility and notable anti-inflammatory activity, as evidenced by its robust suppression of TNF-α and IL-6 production, as well as a moderate but statistically significant reduction in NO levels. Further antioxidant evaluations demonstrated that BSP60 possesses strong free radical scavenging activity against ABTS⁺^•^ and O_2_^•-^ radicals, indicating a capacity to mitigate oxidative stress. Collectively, these findings establish a clear link between the fine structure of *Bletilla striata* glucomannan and its functional properties, highlighting BSP60 as a structurally defined, bioactive polysaccharide with promising potential for biomedical and cosmetic applications.

## Data Availability

Data will be made available on request.
